# The Convention of Medical Teachers

**Published:** 1859-06

**Authors:** 


### THE CONVENTION OF MEDICAL TEACHERS.

We publish the proceedings of the *farce* which recently came off at Louisville, and which was to have been the first act in the *tragedy*, the consummation of which was to have been presented in the American Medical Association.

We have not the time at present to exhibit, as we shall do perhaps, upon some future occasion, the explanation of this new phase of medical intrigue. Suffice it to say for the present, that the authors of the movement—those with whom *this agitation* originated, and by whom it is kept up, were not present or represented, save through their puppets. The truth in regard to this whole question, is, that the mass of the medical profession, singularly enough called "laity," have had no agency in the production of the excitement of the last three or four years upon this subject, and the attempt to *force* them into action upon it, has most *signally failed.*

